# A Novel Approach for Assessing the Fatigue Behavior of PEEK in a Physiologically Relevant Environment

**DOI:** 10.3390/ma11101923

**Published:** 2018-10-10

**Authors:** Mirco Peron, Jan Torgersen, Filippo Berto

**Affiliations:** Department of Industrial and Mechanical Engineering, Norwegian University of Science and Technology, Richard Birkelands vei 2b, 7034 Trondheim, Norway; jan.torgersen@ntnu.no (J.T.); filippo.berto@ntnu.no (F.B.)

**Keywords:** fatigue assessment, PEEK, strain energy density, theory of critical distances, NSIF, biomaterial, PEEK implants

## Abstract

In recent years, the need of surgical procedures has continuously increased and, therefore, researchers and clinicians are broadly focusing on the development of new biocompatible materials. Among them, polyetheretherketone (PEEK) has gained wide interest in load-bearing applications due to its yielding behaviour and its superior corrosion resistance. To assure its reliability in these applications where notches and other stress concentrators weaken implants resistance, a design tool for assessing its tensile and fatigue behaviour in the presence of geometrical discontinuities is highly claimed. Herein, a new fatigue design method based on a local approach is proposed for PEEK implant, and the results are compared with those obtained using the two main biomaterial design approaches available in literature, i.e., the theory of critical distances (TCD) and the notch stress intensity factor (NSIF) approach. To this aim, previously published datasets of PEEK-notched specimens are used, and the proposed method is reported to provide more accurate results and to be robust for different notch geometries.

## 1. Introduction

In recent decades, due to the increased lifetime in the world population, the necessity of implant devices is continuously increasing [1]. An ever-growing range of devices include pacemakers, cardiovascular stents, defibrillators, neural prosthetics and drug delivery systems [2,3,4,5], but load-bearing implants rule the bio-devices market and attract large scale researcher efforts since they pose a significant challenge [4,6,7]. For example, artificial limbs have to fit perfectly to the patient specific site of interest and have to resemble the mechanical properties of the bone to avoid problems, such as stress shielding [8,9,10]. Moreover, load-bearing implants are intermittently stressed due to weight and activity. For example, prostheses implanted to the lower extremities of the body have to withstand loads several times heavier than body weight [11,12,13]. 

Biologically-attractive implant design might cause challenging mechanical conditions leading to fatigue and early failure. For example, engineered porosity is suitable for enhanced osseointegration and an ideal approach for specifically tailoring mechanical properties to fit to the biological site of interest [14]. Yet, such designs lead to stress concentrators. Further, engineered surface roughness has been shown to favour cell adhesion and growth [15,16,17,18]. However, rough surfaces also lead to crack initiation. These competing requirements are aggravated due to the corrosive environment in the human body, leading to effects such as corrosion fatigue [19,20]. Yokoyama et al. [21] studied a titanium occlusal screw that failed after three years of implantation, and they found the fatigue crack to nucleate at the root of the thread. Moreover, Chao and Lopez reported that corrosion fatigue was responsible of the failure of almost 90% of Ti-6Al-4V hip prosthesis [22]. In this challenging scenario, the demand for a robust design tool for the prediction of implant fatigue life is high, especially in the presence of stress raisers. Leveraging developments in the engineering field. 

Many researchers have attempted to predict implant behaviour under dynamic loads of differently-notched components using linear elastic fracture mechanics (LEFM) theory and, in particular, the notch stress intensity factors (NSIFs) [23]. The NSIF approach predicts the fatigue life of notched components comparing the NSIF at which the implant is subjected with a reference fatigue curve determined by testing samples weakened by the same notch geometry [24,25]. However, the need of evaluating accurately the stress field ahead of the geometrical discontinuities to correctly perform the fatigue assessment has limited its development, leading to time-consuming stress field analyses. In addition, the geometry dependence of the NSIF-based criteria represents another drawback of this approach. Their units is in fact MPa(m)*^β^*, where the exponent *β* depends on the notch opening angle, according to the expression *β* = 1 − *λ*_1_, where *λ*_1_ is the Williams’ eigenvalue [26]. 

The geometry dependence implies the necessity of ad hoc experimentally determined curves to use the NSIF method, representing thus a complexity in its use. The theory of critical distance (TCD) allows to overcome the geometry-dependence limitation. TCD represents a set of methodologies based on the definition of a material parameter called the critical distance, L. According to the TCD, failure of notched components subjected either to static or cyclic loadings is stated to occur when the stress averaged over a line (line method, LM) or calculated at a certain distance from the notch root (point method, PM) equals the inherent material strength *σ*_0_ or the plane specimen fatigue limit Δ*σ*_0_ depending on whether tensile or fatigue assessment is considered. Kasiri and Taylor [27] applied LM to predict the fracture behaviour of bones weakened by different holes subjected to various loading scenario. 

Although the general trend was well predicted, the results underestimated the fracture stress by 20–30%. In fact, it is generally acknowledged by proponents of critical distance methods that the point and line method are limitation of a more accurate approach that average stresses over a certain volume (or area in 2D problems) in the vicinity of the hot-spot. Taylor [28] thus formalized the so-called area method (AM), where the range of the maximum principal stress is averaged over a semi-circular area. However, although the accuracy of the results increases considering the AM, stress fields ahead of the notch still need to be accurately determined, resulting in high-performance hardware demand. This has been overcome by the strain energy density (SED) approach, according to which failure, in the form of a fatigue crack onset, occurs when the strain energy range $\Delta \bar{W}$ averaged in a control volume of radius *R_C_* ahead of the notch or crack tip reaches its critical value Δ*W_C_*, independently from the notch geometry [29,30,31]. Moreover, the strain energy range can either be obtained analytically, deriving the stress fields ahead of the geometrical discontinuity, or by means of FE analyses with a coarse mesh, being mesh-insensitive [32,33]. This tool has been widely reported to accurately predict the tensile and fatigue behaviour of different notched materials [34,35,36,37,38] and for real component such as steel rollers [39] and its improvements with respect of the TCD methodologies has been reported in details in [40] for Ti-6Al-4V. In addition, it has recently been utilized to predict the fracture behaviour of plastics [41,42,43], including the biocompatible biopolymer polyetheretherketone (PEEK) [44], an emerging material due to its yielding behaviour and its superior corrosion resistance, also in physiologically relevant environments [4,45,46,47,48]. Due to its outstanding properties, PEEK, besides being studied as a substitute material for metals in gear wheels and food processing [49], has also gained interest in biomedical applications such as spinal cages [50] and dental implants [51,52]. PEEK is in fact characterized by an in vitro bone stimulation capacity comparable to that of cp Ti grade 1 [53] and by an elastic modulus closer to that of human bones compared to that of metals, as reported in [9]. As a consequence, the bone resorption by the stress shielding effect induced by PEEK implant is far lower than that induced by titanium and zirconia implants, as assessed by Lee et al. [54]. In addition, they reported un-notched PEEK components to be able to withstand static and cyclic loadings comparable to those deriving by bites in anterior dentitions. Due to the increasing interests in PEEK for biomedical applications, a reliable failure criterion for this material is highly required, especially when weakened by notches, and the authors have herein decided to assess the reliability of the SED approach in predicting the fatigue behaviour of PEEK materials in a physiologically relevant environment. This poses two challenges since this tool was never used for fatigue of polymers and the effects of corrosion could yet not be revealed. For our reference, we employ the experimental data of Sobieraj et al., who investigated PEEK tensile and fatigue behaviour in a physiologically relevant environment in the presence of stress concentrators [55,56]. Their fatigue data were here analysed in terms of SED to assess the reliability of the method as a fatigue property prediction model for biomaterials. Moreover, these data have been analysed also by means of NSIF and LM approaches to highlight the SED advantages.

## 2. Material and Methods

**Figure 1:**
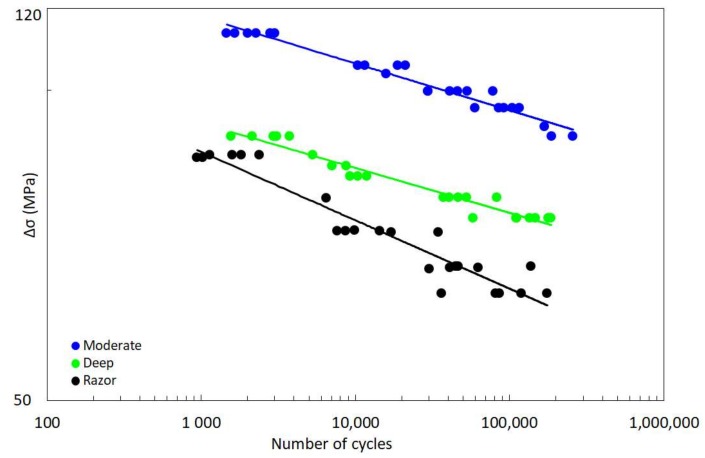
Experimental fatigue data. Modified from Sobieraj et al. [55].

Sobieraj et al. [55] aimed to determine the S-N behaviour of PEEK in the presence of stress concentrators and to estimate the duration of initiation versus propagation in a physiologically relevant environment. They preconditioned the specimens for 8 weeks and then carried out fatigue tests in a 37 °C phosphate-buffered saline (PBS) bath. Cylindrical specimens with a 8 mm outer diameter were manufactured and weakened by three different type of notches, i.e., two circumferentially U-notched specimen geometries, both with a 6 mm inner diameter, but different notch radii being 0.9 mm for the specimens labelled as moderate and 0.45 mm for those labelled as deep, and a circumferentially razor-grooved dog-bone. For more details about the specimen geometries the reader could refer to [55]. Tension-tension fatigue tests were carried out aiming to assess the fatigue behaviour with number of cycles to failure ranging from 1000 to 100,000 at a frequency of 2 Hz with a load ratio equal *R* ≤ 0.02. The fatigue data, shown in Figure 1, were fitted by Sobieraj et al. [55] by means of the S-N Basquin relationship [57]:

(1) $$\Delta \sigma =AN^{d}$$

where Δ*σ* is the stress range, *N* is the number of cycles to failure, and *A* and *d* are constants, as reported in Table 1.

Moreover, Sobieraj et al. estimated the number of cycles *N_p_* necessary to propagate a crack from an initial length *a_i_* to a final length *a_f_* utilizing the Paris relationship for the cyclic crack growth. For further details the reader should refer to [55]. From these analyses the initiation phase was reported to be predominant respect to the propagation phase (in almost all the samples the initiation phase was higher than the 95% of the whole fatigue life). This allows the authors to analyse the fatigue data by means of the SED approach. In fact, the main requirement of this approach for a reliable fatigue strength prediction lies on a predominance of the life spent for crack initiation in the whole fatigue life. In fact, the SED approach has been formalized to predict the onset of a fatigue crack and its extension to the complete failure assessment is thus related to a predominance of the life spent for crack initiation in the whole fatigue life. In implants design, these requirements does not represent limitations; crack onset tends to be avoided since it influences the mechanical behaviour of the implants, modifying its compliance and causing a non-uniform deformation, leading to an incorrect implant functioning.

## 3. Results and Discussion

**Figure 2:**
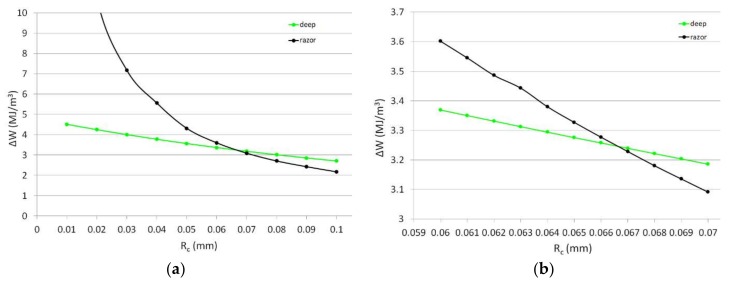
Determination of the SED critical parameters. The value of the critical radius *R_C_* has been varied with a step of 0.01 mm (**a**) and then with a finer step of 0.001 mm (**b**).

The application of the SED approach requires the determination of the so-called critical SED parameters, i.e., the critical radius of the control volume *R_C_* and the critical value of the strain energy density range Δ*W_C_*. In [58] simple analytical formulations for their determination have been proposed and are reported in Equations (2) and (3) for the former and the latter, respectively:

(2) $$R_{c}={\left(\frac{\sqrt{2e_{1}}\Delta K_{1,A}}{\Delta \sigma_{0}}\right)}^{\frac{1}{1-\lambda_{1}}}$$

(3) $$\Delta W_{c}=\frac{\Delta \sigma_{0}^{2}}{2E}$$

where Δ*σ*_0_ is the fatigue limit of the material without geometric singularities, Δ*K*_1*A*_ is the Notch Stress Intensity Factor (NSIF) fatigue threshold, *λ*_1_ is Williams’ eigenvalue for mode 1 [26], *e*_1_ is a parameter dependent on the notch opening angle 2*α*, on the hypothesis considered (plane strain or plane stress) and on Poisson’s ratio *ν* (the reader should referred to [58] for *e*_1_ formulation) and *E* is the Young’s modulus of the material. In plane problems the control volume becomes a circular sector or a circle, centred at the notch tip or at the crack tip depending on whether sharp V-notches or cracks are considered. For a blunt V-notch or a U-notch the circular sector is no longer centred at the crack tip, but moved back of a quantity *r*_0_ dependent on the notch opening angle. For further details the reader should refer to [44]. Being neither NSIF fatigue threshold nor fatigue limit of the plain material available, Equations (2) and (3) cannot be applied to determine the critical SED parameters, and an alternative approach needs thus to be used. Leveraging on the possibility offered by finite element (FE) codes like Ansys^®^ (Canonsburg, PA, USA) to easily evaluate the strain energy within the control volume, the determination of the SED critical parameters can be performed varying the value of the radius of the control volume until the SED values for two different specimen geometries at the same number of cycles are equal, corresponding to the critical SED range value for that number of cycles, as done in [59,60]. Circumferentially razor-grooved dog-bone and U-notched specimen with a notch root radius of 0.45 mm have been modelled in Ansys^®^ (Canonsburg, PA, USA), and the simulations have been carried out for different values of *R_C_*, ranging from 0.01 to 0.1 mm, with a step of 0.01 mm (Figure 2a). Then, in the range between 0.06 and 0.07 mm where the difference between the SED values for the two geometries at 150,000 cycles were lower, the simulations have been repeated with a step of 0.001 mm for the critical radius until a correspondence was found, resulting in *R_C_* = 0.067 mm and Δ*W_C_* = 3.23 MJ/m^3^ at 150,000 cycles (Figure 2b).

Axisymmetric linear elastic 2D analyses using the eight-node axisymmetric element plane 83 have been carried out, with an elastic modulus of 3500 MPa and a Poisson’s ration of 0.36 [56]. In addition, being the SED approach mesh-insensitive, a coarse mesh at the crack tip (element size of about 10^−1^ mm) has been adopted. Further details about mesh pattern and boundary conditions can be found in [44].

**Figure 3:**
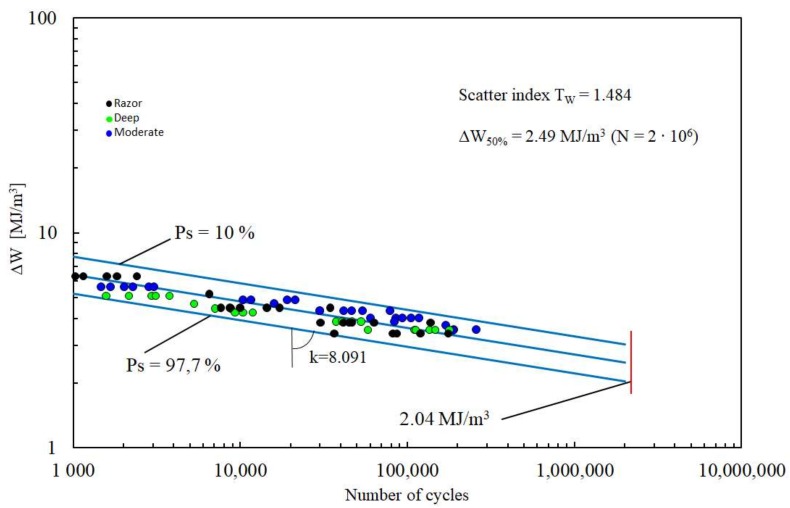
Synthesis of PEEK fatigue data by means of SED approach.

Once the critical radius was determined, the fatigue data shown in Figure 1 were analysed in terms of SED range. FE analyses were carried out considering the experimental stress range as the applied load in the simulations, determining the corresponding SED range value used to obtain the fatigue curve shown in Figure 3.

**Figure 4:**
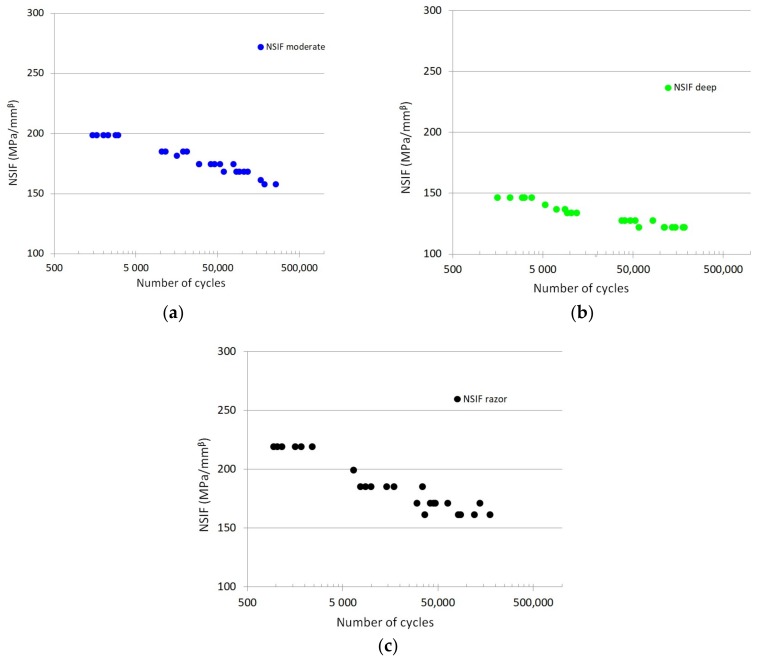
Fatigue NSIF-N curves for specimens labelled as moderate (**a**), deep (**b**) and razor (**c**), where *β* is equal to 0.5.

The fatigue results felt within a single narrow scatter band, with an inverse slope *k* = 8.091 and a strain energy density range referred to 2 million loading cycles and to a probability of survival of 50%. In fact, the scatter index *T_W_*, related to the two curves with probabilities of survival *P_s_* = 2.3% and 97.7%, is 1.484. *T_W_* = 1.484 becomes equal to 1.22 when reconverted to an equivalent local stress range being the SED proportional to the square of the stress (*T_σ_* = $\sqrt{1.484}$ = 1.22). Comparing Figure 1 with Figure 3 it can be noted how the SED approach is able to summarize the fatigue data obtained from different notch geometries within a single narrow scatter band, whereas the S-N curves show a different behaviour for each notch geometry. This represents a milestone in the design of biomedical implants; once a ΔW-N curve had been obtained for any notch geometry, it can be considered as the SED fatigue master curve, which can be used as reference for every kind of notch geometries weakening the components. As it is evident from the just reported procedure, the advantages of SED approach lie on its simplicity and rapidity-to-use that could render it a breakthrough in the design procedures when compared to the methods so far used. Compared with the NSIF approach for example, the SED criterion inherits its simplicity (the comparison with a reference fatigue curve) but it overcomes the NSIF approach main limitation of the geometry dependency, i.e., the need of a reference curve for each notch geometry (Figure 4). This advantage of the SED derives from its scalar nature, being its unit MJ/m^3^ regardless of the notch opening angle, whereas the NSIF unit depends on the notch opening angle as described in the introduction.

**Figure 5:**
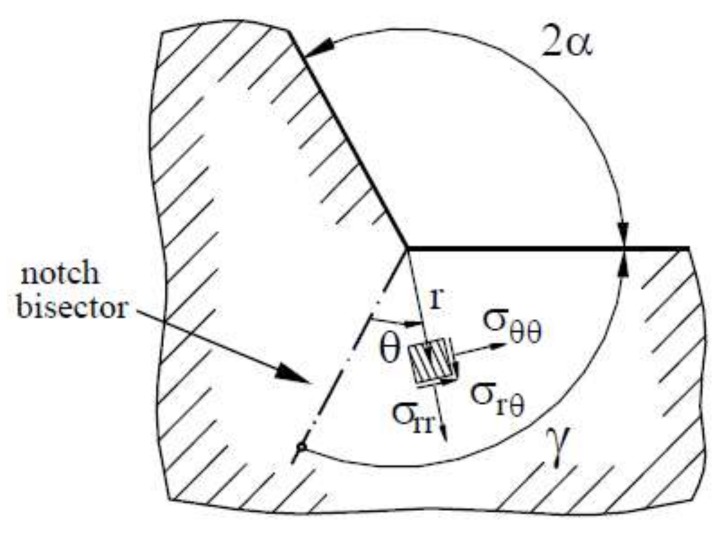
Polar coordinate system centred at the notch tip. Reproduced with permission from [58], Springer, 2001.

NSIFs have in fact been defined according to Gross and Mendelson [61]:

(4) $$K_{1}=\sqrt{2\pi }\underset{r\to 0}{\lim}r^{\left(1-\lambda_{1}\right)}\left[\sigma_{\theta \theta }\left(r,\theta =0\right)\right]$$

where *r* and *θ* is the radial and angular coordinate of a polar coordinate system centred at the notch tip and *σ_θθ_* is the stress components according to the coordinate system (Figure 5).

Stress distribution has been found using FE analyses leveraging on the aforementioned FE models, with the only exception that a fine mesh is required to be adopted, being the results widely known to be mesh-sensitive. In addition, when U-notches and blunt V-notches are considered, the NSIF approach is not valid anymore and a generalized NSIF has to be adopted [62]. In fact, when the notch root radius is different from zero, the characteristic, singular, sharp-notch field diverges from the rounded-notch solution very next to the notch and as the notch root radius *ρ* increases, the usefulness of the NSIF rapidly decreases. In these cases the advantages of SED approach are even larger being the generalized NSIF dependent not only on the opening angle, but also on the notch depth and radius, being defined as:

(5) $$K_{\rho ,1}=\sqrt{2\pi }r^{1-\lambda_{1}}\frac{{\left(\sigma_{\theta }\right)}_{\theta =0}}{1+\tilde{\omega_{1}}{\left(\frac{r}{r_{0}}\right)}^{\mu_{1}-\lambda_{1}}}$$

where *σ_θ_* is the stress at a distance *r* from the notch, while $\tilde{\omega_{1}}$ and *µ*_1_ are functions of notch depth and radius and are reported in [62]. In fact, considering the geometries herein studied, a unique reference curve for the U-notched specimens (labelled here as moderate and deep) cannot be obtained (Figure 4a,b), precisely because the generalized NSIFs depend on notch depth and radius.

Moreover, the SED approach allows to overcome the other NSIF approach drawback, i.e., the need of a fine mesh to accurate describe the stress field. In fact, when the goal of the finite element analysis is to obtain the strain energy density, coarse meshes are sufficiently accurate since the results are derived directly from nodal displacements [63]. This also allows to overcome the main limitation of the line method. To reliably predict the fatigue failure of components, in fact, the method requires the stress distribution ahead of the stress raiser to be accurately determined and, thus, a fine mesh is required in FE analyses. The LM states the failure, regardless of the specimen geometry, to occur when the first principal stress range Δ*σ*_1_ averaged along a distance equal to twice the critical distance *L* reaches a characteristic value of the material, i.e., the plain-specimen fatigue limit Δ*σ*_0_ if the failure at 2 × 10^6^ cycles is considered:

(6) $$K_{1}=\sqrt{2\pi }\underset{r\to 0}{\lim}r^{\left(1-\lambda_{1}\right)}\left[\sigma_{\theta \theta }\left(r,\theta =0\right)\right]$$

where *L* is the critical distance defined as:

(7) $$L=\frac{1}{\pi }{\left(\frac{\Delta K_{th}}{\Delta \sigma_{0}}\right)}^{2}$$

where Δ*K_th_* is the fatigue limit of a cracked specimen, i.e., the stress intensity factor fatigue threshold. Again, being not known neither the fatigue limit neither for plain nor for cracked specimen, Equations (6) and (7) cannot be used to determine the critical TCD parameters, i.e., *L* and Δ*σ*_0_. To overcome this limitation, the authors has leveraged on the Point Method (PM), which states the failure to occur when the first principal stress range Δ*σ*_1_ evaluated at a distance equal to half the critical distance *L* reaches the plain-specimen fatigue limit Δ*σ*_0_ if the failure at 2 × 10^6^ cycles is considered:

(8) $$\Delta \sigma_{1}\left(r=\frac{L}{2}\right)=\Delta \sigma_{0}$$

**Figure 6:**
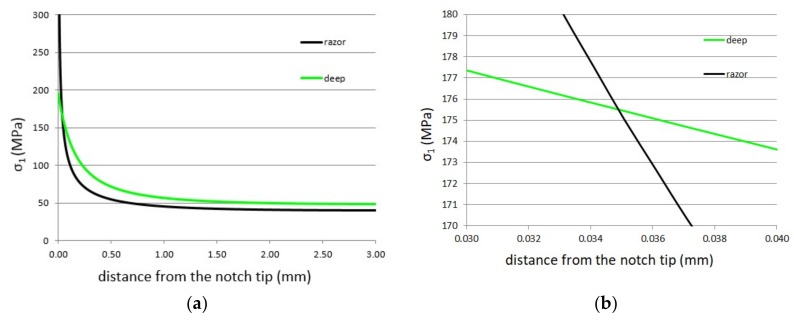
First principal stress distribution ahead of the notch tip for of “razor” and “deep” specimen (**a**); detail of the first principal stress distribution to determine the critical TCD parameters according to the point method, where the intersection occurs at half the value of the critical distance *L* (**b**).

Thus, assessing the stress fields ahead of the notch for both circumferentially razor grooved dog-bone and U-notched specimen with a notch root radius of 0.45 mm by means of Ansys^®^ code (Canonsburg, PA, USA), the TCD parameters can be estimated, leading to a critical distance *L* and a plain-specimen fatigue limit Δ*σ*_0_ of 0.07 mm and 175.5 MPa, respectively (Figure 6).

**Figure 7:**
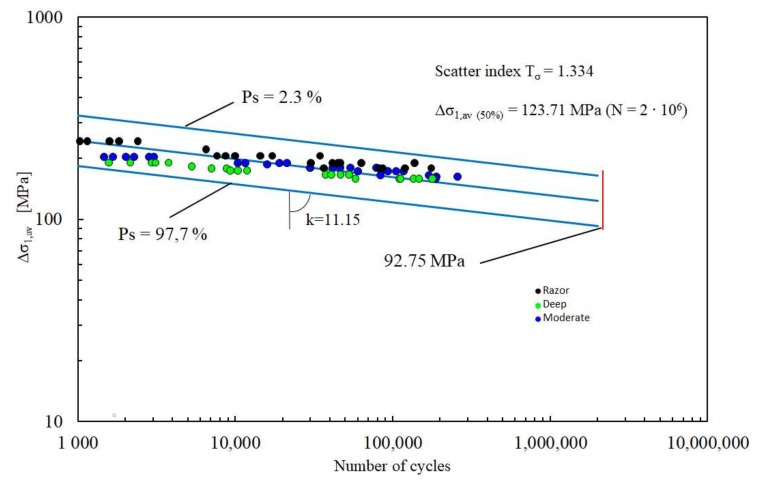
Synthesis of PEEK fatigue data by means of the line method.

FE models were the same used for NSIF analyses. Once determined the TCD critical parameters, the fatigue data obtained in [55] have been analysed by means of LM, leading to a single fatigue curve able to summarize the experimental data for all the different notch geometries (Figure 7).

The curve has been obtained considering the failure stress range as the applied load in the FE analyses, averaging the first principal stress range distribution along a length equal to twice the critical distance *L*, and reporting the results as a function of the corresponding fatigue life. Although the capability to summarize the fatigue data of different notch geometries within a single scatter band corresponds to that of SED approach, the results are characterized by a scatter higher than that obtained with SED approach, being *T_σ_* = 1.33 instead of 1.22. Summarizing, the SED approach is characterized by two main advantages when compared to LM, providing a narrow scatter band in the assessment of the fatigue data and requiring the FEM analyses lower computational efforts being the SED criterion mesh-insensitive. 

These developments in assessing the fatigue strength of PEEK in a physiologically relevant environment provided by the SED approach open the road towards an increasing use of this material in several orthopaedic applications, among which femoral component of total hip replacements, bone anchors and spinal implants [64].

## 4. Conclusions

The strain energy density (SED) approach has revealed in the past to greatly predict both the tensile and fatigue behavior of several notched metals parts. Herein this approach has been extended to the fatigue behaviour of PEEK, with satisfactory results. The authors have utilized a previously supplied dataset [55] where different notch geometries in a physiologically relevant environment have been tested, revealing how PEEK fatigue behaviour is notch-sensitive. Compared to the two main methods used in the fatigue assessment of bio-materials, i.e., the NSIFs approach and the line method, it has shown the following advantages:

- With respect to NSIFs approach, SED criterion has been shown to summarize fatigue data for different notch geometries within a single narrow scatter band.
- Compared to LM, SED approach is characterized by a lower scatter, i.e., *T_σ_* = 1.33 and 1.22 for LM and SED criterion, respectively.

In addition, both NSIFs and LM require high computational efforts being a fine mesh required ahead of the notch tip, whereas the SED approach has revealed to be mesh-insensitive.

This work introduces a milestone in the design of PEEK biomedical devices, providing a simple and rapid method for assessing the fatigue strength, overcoming the drawbacks of the other methods, and opening the road towards the use of this approach in corrosive environments.

## Figures and Tables

**Table 1:** S-N Basquin relationship constants. Data taken from [55].

Parameters	Moderate	Deep	Razor
***A* (MPa)**	152	120	131
***d***	−0.043	−0.043	−0.063
***R*^2^**	0.95	0.90	0.92
